# Porcine milk-derived exosomes promote proliferation of intestinal epithelial cells

**DOI:** 10.1038/srep33862

**Published:** 2016-09-20

**Authors:** Ting Chen, Mei-Ying Xie, Jia-Jie Sun, Rui-Song Ye, Xiao Cheng, Rui-Ping Sun, Li-Min Wei, Meng Li, De-Lin Lin, Qing-Yan Jiang, Qian-Yun Xi, Yong-Liang Zhang

**Affiliations:** 1 National Engineering Research Center For Breeding Swine Industry, Guandong Provincial Key Laboratory of Agro-Animal Genomics and Molecular Breeding, South China Agricultural University, 483 Wushan Road, Guangzhou 510642, China

## Abstract

Milk-derived exosomes were identified as a novel mechanism of mother-to-child transmission of regulatory molecules, but their functions in intestinal tissues of neonates are not well-studied. Here, we characterized potential roles of porcine milk-derived exosomes in the intestinal tract. *In vitro*, treatment with milk-derived exosomes (27 ± 3 ng and 55 ± 5 ng total RNA) significantly promoted IPEC-J2 cell proliferation by MTT, CCK8, EdU fluorescence and EdU flow cytometry assays. The qRT-PCR and Western blot analyses indicated milk-derived exosomes (0.27 ± 0.03 μg total RNA) significantly promoted expression of CDX2, IGF-1R and PCNA, and inhibited p53 gene expression involved in intestinal proliferation. Additionally, six detected miRNAs were significantly increased in IPEC-J2 cell, while *FAS* and *SERPINE* were significantly down-regulated relative to that in control. *In vivo*, treated groups (0.125 μg and 0.25 μg total RNA) significantly raised mice’ villus height, crypt depth and ratio of villus length to crypt depth of intestinal tissues, significantly increased CDX2, PCNA and IGF-1R’ expression and significantly inhibited p53′ expression. Our study demonstrated that milk-derived exosomes can facilitate intestinal cell proliferation and intestinal tract development, thus giving a new insight for milk nutrition and newborn development and health.

The development of the gastrointestinal tract (GIT) progresses through the prenatal and postnatal periods. During postnatal development, the GIT undergoes profound growth, morphological changes and functional maturation^1^. A close relationship exists between the degree of maturation and absorptive functions of the intestine. In the neonatal intestine, nutrient transport occurs along the whole crypt–villus axis, whereas in the adult intestine absorption of nutrients is shifted to the upper part of the villi^2^. Therefore, in mammalian nutrition, the GIT is responsible for the first physiological step of bringing nutrients to the body’s cells and plays a crucial role in the regulation of the development and health of infants^3^. The unique composition of breast milk may provide factors that stimulate developmental changes of the neonatal intestine that occur following birth^4^.

After birth, the neonatal GIT is stimulated chiefly by enteral nutritive and non-nutritive (biologically active) substances from colostrum and milk. Nutrition is a critical determinant in the functional growth and maturation of the GIT^5^. Thus, malnutrition in the postnatal period may restrict the morphologic and biochemical development of the GIT^6^. In milk, regulation of proteins, lipids and nucleotide synthesis to orchestrate anabolism, cell growth and proliferation occurs by activating the mTORC1 signaling pathway^7^. Hormones and growth-promoting peptides in milk, such as insulin, cortisol, epidermal growth factor (EGF) and insulin-like growth factor I (IGF-I), have been found to play a role in postnatal GIT development in newborns^1^. Differences in colostrum and milk composition between species and responses to milk-borne bioactive components may be expected due to ontogenic development of tissues and organs^8^.

Exosomes are nanosized endosome-derived membrane vesicles (40–100 nm in diameter), which form from inward budding of early endosomes to become multivesicular endosomes (MVB) containing intralumenal vesicles^9^. Exosomes contain mRNA, microRNA (miRNA), DNA, proteins and lipids that can be transferred to cells to confer new functions or cell signaling events^10^,^11^,^12^, which are involved in cell-cell communication via the functionally-active cargo^13^. Exosomes are present in breast milk and are packed with abundant immune-related proteins (such as MHC class II, CD86 and the tetraspanin proteins, CD63 and CD81)^10^, as well as miRNAs. They were proposed to have the potential to influence the immune system of infants^14^,^15^. Human milk exosomes are capable of surviving digestion and being taken up by enterocytes into the nucleus and may affect gene expression through exosomal miRNAs^16^ or enter the infant’ systemic circulation to exert the functions of tissue-specific immunoprotective and development^17^. Bovine milk-derived extracellular vesicles (BMEVs) have been shown to be taken up by murine macrophage RAW264.7 cells, splenocytes and intestinal cells *in vitro*, and to reduce the serum levels of MCP-1 and IL-6 in splenic cells^18^.

Even though milk-derived exosomes are known to be resistant to a harsh environment and are taken up by multiple cells, including intestinal cells, whether they regulate intestinal cell proliferation and intestinal tract development remains unclear. Therefore, based on our previous exploration of miRNAs, mRNAs and proteins in porcine milk^19^, the KEGG pathway analysis of potential targets of top 10 miRNAs in porcine milk were enriched in Notch signaling pathway which could play an early critical role in cell fate determination and later roles in the regulation of cell proliferation and differentiation^20^. Otherwise, the Notch signaling pathway could participate in regulation of human cervical cancer and mammary gland progression^21^,^22^. Thus, we hypothesized that porcine milk-derived exosomes are capable of regulating intestinal cell proliferation and intestinal tract development. We hope this study could provide a new evidence for the regulation of intestine tract development from breastmilk exosomes to offspring.

## Results

### Milk-derived exosomes promoted proliferation of IPEC-J2 cell

**Figure 1:**
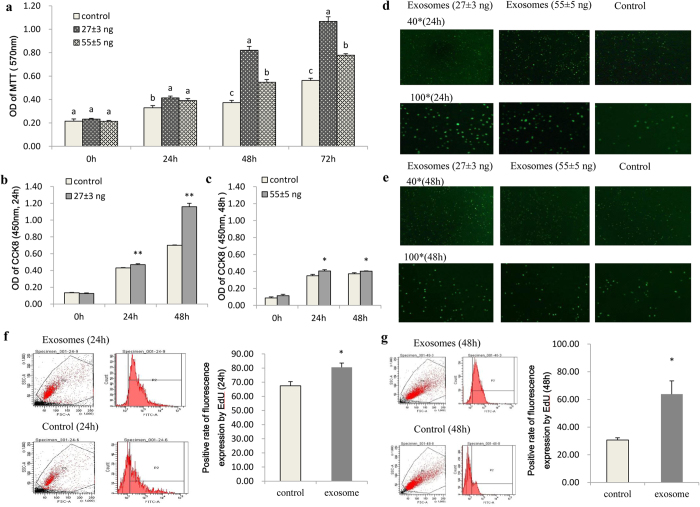
IPEC-J2 cell proliferation detected by different methods. (**a**) Both 27 ± 3 ng and 55 ± 5 ng total RNA groups showed significantly improved OD values in the MTT assay, but the effect of the 27 ± 3 ng treatment group was more obvious (n = 10). (**b,c**) Effects of 27 ± 3 ng and 55 ± 5 ng total RNA treatment groups. The 27 ± 3 ng treatment group showed a significantly improved OD value (*P* < 0.01) in the CCK8 compared with the 55 ± 5 ng treatment group (n = 10). (**d,e**) Fluorescence intensity of IPEC-J2 cells after treatment with 27 ± 3 ng and 55 ± 5 ng total RNA for 24 h and 48 h. The 27 ± 3 ng treatment group showed greater fluorescence intensity by EdU fluorescence microscopy (n = 6). (**f,g**) Positive ratio of the 0.27 ± 0.03 μg total RNA (6-well plate) treatment group at 24 h and 48 h, respectively. The positive ratio significantly increased up to 80% after treatment for 24 h and 67% at 48 h by Edu flow cytometry (n = 6, 24 h).

IPEC-J2 cells were seeded at a density between 4,000 to 12,000 cells per/well (96-well plate), and their growth peaked between 24 h to 72 h after seeding (see Supplementary Figure 1). Porcine milk-derived exosomes containing 27 ± 3 ng and 55 ± 5 ng total RNA were then added to the IPEC-J2 cell culture, followed by analysis using the MTT assay at 0, 24, 48 and 72 h post treatment. The MTT results showed that treatment with porcine milk exosomes could significantly raise the OD value at 24, 48 and 72 h, and the 27 ± 3 ng total RNA group had the most significant effect (*P* < 0.01) (Fig. 1a).

In the CCK8 assay, porcine milk exosomes containing 27 ± 3 ng and 55 ± 5 ng total RNA significantly improved the OD value of CCK8 compared with the control group, and the 27 ± 3 ng total RNA group showed a more efficient effect than the 55 ± 5 ng total RNA group (Fig. 1b,c). These results were consistent with those of the MTT assay. Furthermore, in the EdU fluorescence assay, the number of fluorescent cells was obviously greater in treatment groups than that in the control group (Fig. 1d,e). As revealed by EdU flow cytometry, milk exosomes of the 0.27 ± 0.03 μg total RNA group (6-well plate) significantly increased the positive fluorescence rates up to 80% at 24 h (Fig. 1f) and 67% at 48 h (Fig. 1g) post treatment, which were greater than that of the control group (Supplementary Figure 2).

### Effect on proliferation-related gene’s expression by milk-derived exosome

**Figure 2:**
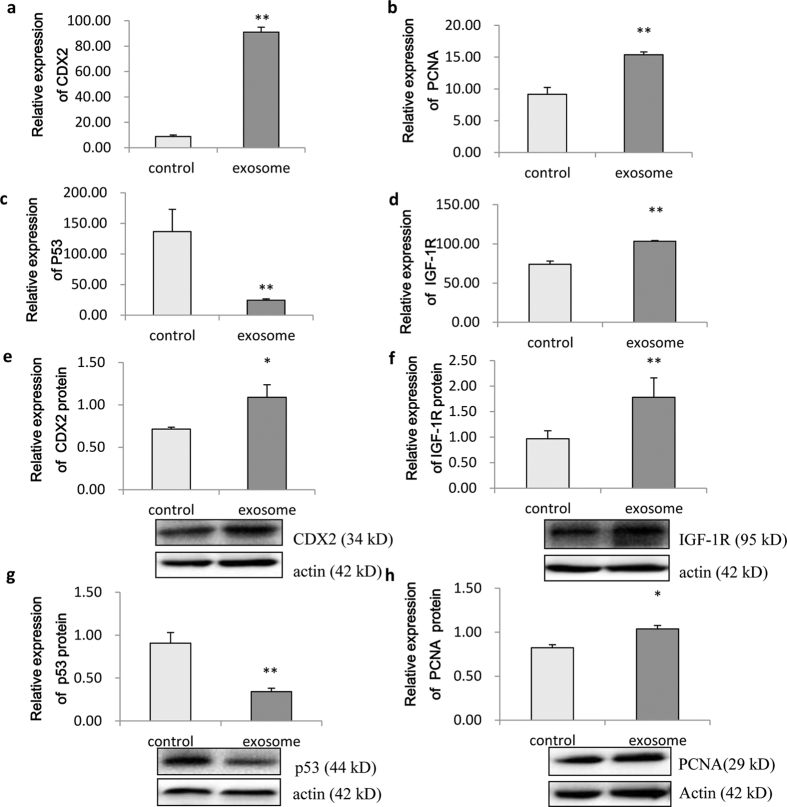
Expression of proliferation-related genes and proteins in IPEC-J2 cells. (**a–d**) Expression of *CDX2*, *PCNA*, *p53* and *IGF-1R* mRNAs, respectively. *CDX2*, *PCNA* and *IGF-1R* levels were significantly improved, while *p53* was inhibited in the 0.27 ± 0.03 μg total RNA treatment group (*P* < *0.01*, n = 6). (**e–h**) Expression of CDX2, IGF-1R, p53 and PCNA proteins, respectively. CDX2, IGF-1R and PCNA were increased significantly, while p53 was inhibited significantly (*P* < *0.01*) (n = 6).

Analysis by qRT-PCR revealed that porcine milk-derived exosomes significantly improved the expression of *CDX2, proliferating cell nuclear antigen (PCNA*) and *IGF-1R* genes (Fig. 2a,b,d) and inhibited the expression of *p53* (Fig. 2c) in IPEC-J2 cells. Similarly, Western blotting showed significantly increased protein expression of CDX2, PCNA and IGF-1R (Fig. 2e,f,h) and significantly inhibited expression of p53 (Fig. 2g, *P* < 0.01). Those changes in protein markers of cell proliferation are fully consistent with the increased cell proliferation observed above.

### Milk-derived exosomes changed miRNA concentration and resulted in changing of mRNA level in IPEC-J2 cells

**Figure 3:**
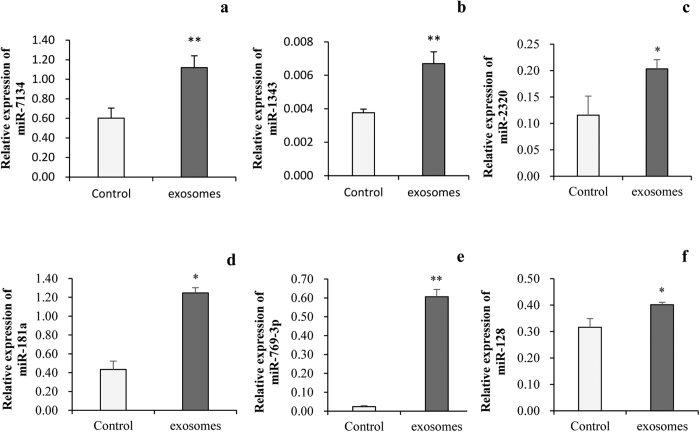
Milk-derived exosome increased miRNA level in IPEC-J2 cells. (**a–f**) Expression of miR-7134, miR-1343, miR-2320, miR-181a, miR-769-3p and miR-128 were significantly increased in IPEC-J2 cell after treated by milk-derived exosomes, respectively (PBS as the control, n = 6).

**Figure 4:**
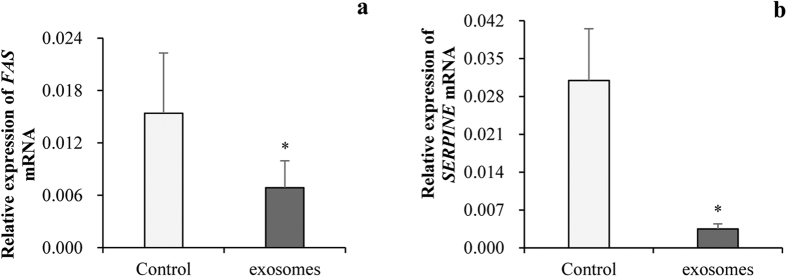
Milk-derived exosome decreased *FAS* and *SERPINE* mRNA expression in IPEC-J2 cell. (**a,b**) Expression of *FAS* and *SERPINE* mRNA were significantly decreased after treatment by milk exosome, respectively (PBS as the control, n=6).

To testify if miRNAs in milk-derived exosomes could enter into IPEC-J2 cells, we determined the level of miR-7134, miR-1343, miR-2320, miR-181a, miR-769-3p and miR-128 in IPEC-J2 cells after incubation with exosomes. q-PCR confirmed those miRNAs showed significantly higher level in IPEC-J2 cells after milk-derived exosomes incubation (Fig. 3b,c) relative to that in control, respectively. Bioinformatics analyzed showed the *FAS* was targeted of miR-2320 and miR-181a and *SERPINE* was target of miR-769-3p and miR-128 in p53 signaling pathways^19^. More interestingly, we analyzed the *FAS* and *SERPINE* which are potential targets of milk exosomal miRNAs and involved in p53 pathways and found out mRNA levels of *FAS* (Fig. 4a) and *SERPINE* (Fig. 4b) were down-regulated after being treated with milk exosome. All those results hinted that the milk-derived exosomes could not only be uptake by IPEC-J2 cells, but also regulate gene expression in recipient cells.

### Milk-derived exosomes affected intestinal tract growth in mice

**Figure 5:**
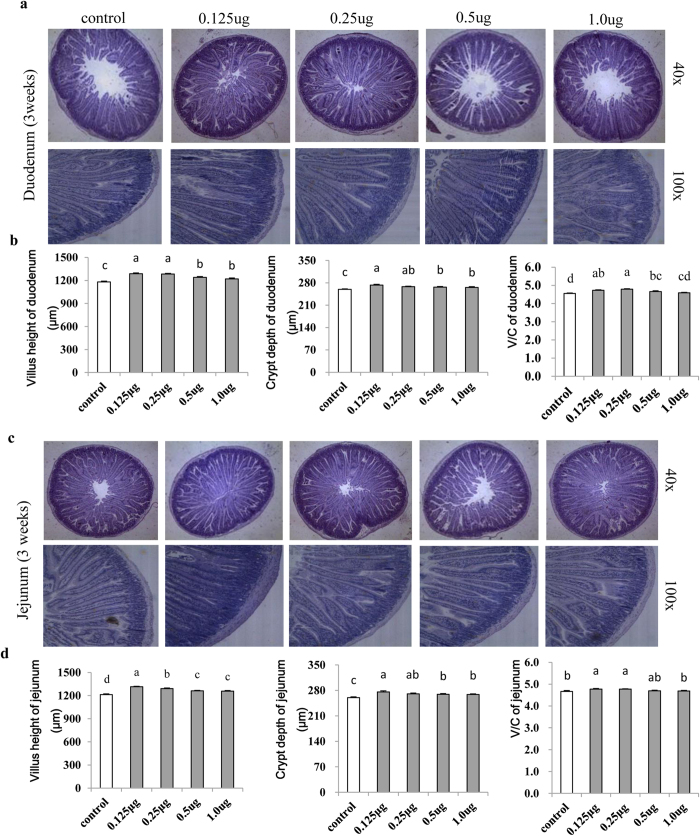
Observation and analysis of intestinal histomorphology (3 weeks). (**a**) The 0.125 μg, 0.25 μg, 0.5 μg and 0.1 μg treatment groups showed improvement in the villus height and crypt depth compared with the control group by microscopy observation of duodenum morphology (n = 6). (**b**) Statistical analysis of the 0.125 μg, 0.25 μg and 0.5 μg treatment groups showed significantly increased villus height, crypt depth and V/C ratio in the duodenum (n = 30). (**c**) The 0.125 μg, 0.25 μg, 0.5 μg and 0.1 μg treatment groups showed improvement in the villus height and crypt depth compared with the control group by microscopy observation of jejunum morphology (n = 6). (**d**) Statistical analysis of the 0.125 μg and 0.25 μg treatment groups showed significantly increased villus height, crypt depth and V/C ratio of the jejunum (n = 30).

To further explore effects of porcine milk exosomes on intestinal tract development, we designed an *in vivo* experiment in mice. Hematoxylin and eosin (H&E) staining of paraffin sections showed that exosome treatments in the 0.125 μg, 0.25 μg, 0.5 μg and 1 μg total RNA groups significantly increased villus height and crypt depth of the duodenum and jejunum relative to the control group, both at 3 weeks (Fig. 5a,c) and 2 weeks (Supplementary Figure 5) post treatment. More interestingly, daily administration of milk exosomes (0.125 μg and 0.25 μg total RNA) significantly increased (*P* < *0.01*) the villus height and crypt depth, and thus improved the V/C ratio (Fig. 5b,d) relative to the control. However, higher dosages of 0.5 μg and 1 μg total RNA resulted in a lower increase of the V/C ratio (Fig. 5b,d) than those of the 0.125 μg and 0.25 μg total RNA groups. Additionally, results at 2 weeks post treatment were similar with those at 3 weeks (Supplementary Figure 5). All of these findings suggested that the milk exosomes are capable of improving the development of the intestinal tract in mice.

### Expression of proliferation-related genes in mouse intestinal tract

**Figure 6:**
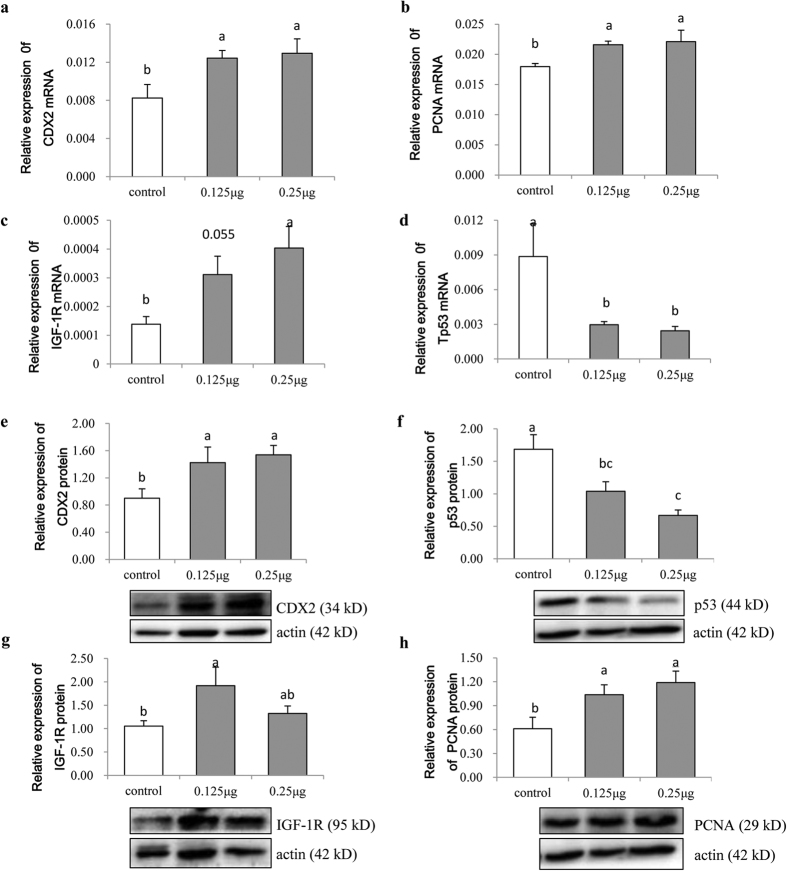
Expression of proliferation-related genes and proteins in intestinal tissue. (**a–d**) Expression of *CDX2*, *PCNA*, *IGF-1R* and *p53* mRNAs, respectively. Expression of *CDX2*, *PCNA* and *IGF-1R* genes significantly increased at different treatment concentrations (0.125 μg and 0.25 μg treatment groups), except for the expression of *IGF-1R* in the 0.125 μg treatment group (*P* = *0.055*). Meanwhile, *p53* significantly decreased (n = 6). (**e–h**) Expression of CDX2, p53, IGF-1R and PCNA proteins, respectively. Except for the IGF-1R protein expression in the 0.25 μg treatment group which did not change significantly, expression levels of other proteins were consistent with those of their corresponding mRNAs (n = 6).

After morphological observation of the mouse intestinal tract, we selected the jejunum tissue (3 weeks) for gene expression analysis. Results showed that 0.125 μg and 0.25 μg of milk-derived exosome RNA significantly improved the expression of *CDX2*, *PCNA* and *IGF-1R* in the jejunum at 3 weeks post treatment (Fig. 6a–c) and significantly decreased the *p53 *expression (Fig. 6d) as revealed by qRT-PCR. More importantly, Western blot analysis also showed increased protein levels of PCNA, IGF-1R and CDX2 (Fig. 6e,g,h) and a decreased p53 protein level (Fig. 6f) in the jejunum. Intriguingly, results above were fully consistent with those of the *in vitro* analysis in IPEC-J2 cells. These findings strongly suggest that milk exosomes promote intestinal cell proliferation by regulating expression of CDX2, PCNA, IGF-1R and p53.

## Discussion

Exosomes are small (30–150 nm) membrane-derived extracellular vesicles that carry miRNAs, proteins and lipids, and mediate intercellular communication. In this study, exposure to porcine milk exosomes resulted in significantly increased proliferation of IPEC-J2 cells, as revealed by analyses using the MTT assay, CCK8 assay and EdU fluorescence and flow cytometry. As a thymidine analogue, EdU can be integrated in cellular DNA during the S-phase of the cell cycle^23^. Thus, the cell fluorescence intensity and the proportion of cells in the S-phase may represent the rate of proliferation. As described in the results, the ratio of positive fluorescent IPEC-J2 cells increased up to 80% by addition of milk-derived exosomes (27 ± 3 ng total RNA group). In the mouse experiment, morphometric analysis showed that the villus height, crypt depth and V/C ratio were significantly increased after treatment with porcine milk exosomes. Intestinal cell proliferation is a primary driver of intestinal growth and development^24^, and the continuous renewal of the intestinal epithelium is closely related to the crypt/villus functional unit. A dynamic system directs this process involving cell generation and migration from the stem cell population, located near the bottom of the crypts, to the extrusion of terminally differentiated cells at the tips of the villi^25^. Indeed, epithelial cells of the different compartments of the crypt–villus axis are characterized by differential properties in regard to cellular proliferation as well as differentiation^26^. Both *in vitro* and *in vivo* experiments in this current study strongly suggest that milk exosomes are able to promote intestinal cell proliferation and digestive tract development.

To further explore how milk exosomes can affect intestinal cell proliferation, expression levels of genes and proteins related to intestinal cell proliferation were resolved by qRT-PCR and Western blotting, respectively. CDX2 is an intestine-specific transcription factor expressed in the nuclei of epithelial cells throughout the intestine, from duodenum to rectum^27^, which is directly involved in intestinal development, differentiation and maintenance of the intestinal phenotype^28^. CDX2 is mostly present in the villi or differentiated cell compartment of the small intestine^29^. PCNA is associated with the cell cycle and functions in both DNA replication and repair^30^. PCNA immunolocalization has been reported to be useful as an index of cell proliferation in normal tissues and lymphoid neoplasms^31^. The IGR-1R downstream Akt/Wnt signaling pathways play a critical role in cell proliferation. A previous report on colon cancer showed that attenuated IGF-1R protein levels could suppress cell proliferation and elevate apoptosis even in the presence of IGF-1 via suppression of IGF-1R/Akt/Wnt signaling pathways and activation of p53^32^. All three of these genes tested in this study were significantly up-regulated by milk exosomes, consistent with their functions.

The p53 protein is the main negative regulator of the cell cycle. Alteration or inactivation of p53, or interactions with oncogene products of DNA tumor viruses that depend on p53, would lead to cancer^33^. p53 controls the expression of the gene encoding the p21 cyclin-dependent protein kinase (CDK) regulator. In normal human cells, p21 is dependent on cyclin, CDK and PCNA, and it controls CDK activity, thereby affecting cell-cycle control^30^. A rapid rise in p53 levels can inhibit cell growth, and tight regulation of p53 function is critical for normal cell growth and development^34^. Meanwhile, Mdm2 is a potent inhibitor of p53, which can block its regulation of target genes and exert anti-proliferative effects^35^. In our results, p53 expression in IPEC-J2 cells and the mouse intestinal tract was significantly inhibited.

How milk exosomes affect gene expression is another topic of interest, which awaits further exploration. As we previously reported, porcine milk exosomes contain hundreds of miRNAs^19^. By bioinformatics analysis, miR-34a, miR-34c, miR-885-3p and miR-885-5p were predicted to play roles in cell fate control and development as related to the Notch signaling pathway^36^. A report in HeLa cells demonstrated that miR-223 targets IGF-1R and suppresses cell proliferation by activation of the downstream PI3K/Akt/mTOR/p70S6K pathway^37^. In ovarian cancer and other cancers, loss of miR-31 can lead to a defect in the p53 pathway^38^. Genes encoding miRNAs in the miR-34 family are direct transcriptional targets of p53, which suppresses tumor formation through integration of multiple transcriptional targets, and miR-34 may act in concert with other effectors to inhibit inappropriate cell proliferation^39^. miR-885-5p leads to the accumulation of p53 protein and activates the p53 pathway, subsequently inhibiting proliferation and interfering with cell cycle progression and cell survival^40^. Although we do not yet have direct experimental evidence to show that milk exosomal miRNAs regulate CDX2, PCNA, IGF1-R or p53, confirming these findings would likely be possible in future studies.

To explore if the milk-derived exosome could be uptake by IPEC-J2 cell and resultantly influence gene expression, we quantitatively detected expression levels of miRNAs and mRNA in IPEC-J2 cells after incubation with milk exsosomes. Six miRNAs detected were significantly elevated after incubation with milk exosome, which indicates miRNA in milk exosome may be uptake by IPEC-J2 cells. Our results are similarly to the previous reports. It was reported that the DCs-derived (dendritic cells) exosomal miR-155 and miR-146a could be transferred to recipient DCs in immune responses^41^. Invasive and non-invasive UBC cell lines derived exosomes specific miRNAs were transferred into cancer cells and tumor-associated fibroblasts (TAFs)^42^. In our study, mRNA of *FAS* and *SERPINE*, who are potential targets of milk exosomal miRNAs^19^ and involved in p53 signaling pathway, were found to be down-regulated due to milk exosome treatment. Similarly, it was reported that pancreatic cancer (PC) derived exosomal miRNAs inhibited mRNA expression of dendritic cells and induced immune tolerance^43^. Many types of cells have been shown to absorb exosomal microRNAs, where they induced post-translational repression of target mRNAs. A recently study reported that the milk exosomal miRNA (miR)-29b and miR-200c could be absorbed by human, meanwhile, mimicking postprandial concentrations of miR-29b and miR-200c in human embryonic kidney 293 cells, and reporter gene activities significantly decreased by 44% and 17%, respectively. These results demonstrated the miRNAs in milk are bioactive food compounds that regulate human genes^44^. Another study revealed the bovine milk exosomes were incorporated into differentiated THP-1 cells then functioned in human cells by containing RNA^45^. Taken together, milk exosome may be uptake by IPEC-J2 cells, and resultantly regulated gene expression in cells.

Milk exosomes are biologically active vesicles. Human milk exosomes are reported to be capable of surviving digestion by treatment with pepsin and pancreatin, and being taken up by enterocytes where they localize to the nucleus and may affect gene expression^16^. The commercial milk-derived extracellular vesicles are extremely stable under degrading conditions, including low pH, boiling and freezing temperatures and easily taken up by murine macrophages *in vitro*, subsequently facilitating T cell differentiation through their packaged bioactive TGF-β^46^. Transport of bovine exosomes also depends on cell and exosome surface glycoproteins in human and rat intestinal cells by endocytosis^47^. Otherwise, bovine milk exosomes are incorporated into differentiated THP-1 cells and affect human cells through their packaged RNAs^45^. Extracellular vesicle-encapsulated miRNAs also cross the intestinal mucosa by processes involving endocytosis and exocytosis^48^. Another study performed on splenocyte cells, RAW264.7 cells and intestinal cells showed that treatment with BMEVs could reduce the serum levels of MCP-1 and IL-6 produced by splenic cells. Via oral gavage, administration of BMEVs to IL-1Ra^−/−^ mice and collagen-induced arthritis mice was shown to result in delayed onset of arthritis and diminished cartilage pathology and bone marrow inflammation; similarly, it diminished the anti-collagen IgG2a levels and was accompanied by reduced splenic Th1 (Tbet) and Th17 (RORγT) mRNA^18^. All these findings showed that milk exosomes could transfer their encapsulated products and exert their functions on target cells.

Furthermore, some studies showed that extracellular vesicles from commercial milk contained several immunomodulating miRNAs and membrane protein CD63. However, the concentrations of miRNAs in milk were significantly reduced during the milk processing^49^, possibly because of that the milk cells and lipid fractions were usually discarded from formula during the industrial milk preparation procedures^17^, and the human milk (HM) cell and HM fat contain more number of miRNAs than peripheral blood mononuclear cells (PMBCs) and plasma, whilst they were with a strong association in human milk^50^. Nonetheless, infant formulas are manufactured from bovine milk, and their RNA concentrations were found to be significantly lower than that in raw milk. Moreover, the quality-of-milk product indicators miR-148a and miR-200c were significantly lower in the extensively hydrolyzed formula than in the standard and follow-on formulas^51^. The decreased miRNAs may be attributed to the disruption of exosome membranes in milk and exposure of miRNAs released from exosomes to milk RNases^49^, suggesting that milk exosomes play an important role in developmental regulation in neonates and their mechanism needs further investigation.

## Conclusion

Our study is the first report on the regulation of intestinal cell proliferation and digestive tract development by milk exosomes. As described in this study, milk exosomes are a new type of regulator in milk which has promising practical applications. As digestive tract development is crucial for the growth and health of newborns, our results will facilitate the goal of providing better nutrition for newborns.

## Materials and Methods

### Sample collection

Fresh porcine milk samples were collected from 10 healthy Landrace female pigs that had been lactating for 1 to 5 days (after parturition) at the pig farm of the South China Agriculture University (Guangzhou, China). Milk samples were frozen immediately and kept at −80 °C until used.

### Preparation of exosomes

Porcine milk exosomes were separated as previously described^19^. Briefly, about 80–100 mL fresh raw procine milk samples were centrifuged at 2000 × *g* for 30 min at 4 °C to remove milk fat globules (MFGs) and mammary gland-derived cells. Defatted samples were then subjected to centrifugation at 12,000 × *g* for 30 min at 4 °C to remove residual MFGs, casein and other debris. From the supernatant, the membrane fraction was prepared by ultracentrifugation at 110,000 × *g* for 2 h using an SW41T rotor (Beckman Coulter Instruments, Fullerton, CA, USA) for three times, and the supernatant was collected as a control for *in vitro* and *in vivo* experiments. The total RNA was then extracted, and the porcine milk-derived exosome concentration was quantified and expressed as μg total RNA/mL in PBS.

### IPEC-J2 cell culture and treatment with porcine milk exosomes

The IPEC-J2 cell line was cultured in Dulbecco’s modified eagle medium (DMEM/Ham’s F-12 [1:1]) (Invitrogen, Life Technologies, Carlsbad, CA, USA) supplemented with 5% fetal calf serum (FCS) (Invitrogen), 5 ug/ mL insulin (Sigma, St. Louis, MO, USA), 5 ng/mL EGF; Peprotech, Rocky Hill, NJ, USA) and incubated at 37 °C with 5% CO_2_. The IPEC-J2 cells were routinely seeded at a density of 0.5 × 10^5 ^mL^−1^ with 10 mL medium in plastic tissue culture flasks (75 cm^2^ Corning, Corning, NY, USA). Cells formed a confluent monolayer within 4 days and then used in experiments^23^.

Cell lines were seeded in 6-well tissue culture plates (9.6 cm^2^/well) at between 2.5 × 10^5^ to 3.0 × 10^5 ^cells per well in a 2 mL volume, and the 96-well culture plates (0.32 cm^2^/well) were seeded with 5 × 10^3^ to 8 × 10^3^ cells per well in a 200 μL volume. All of the seeded cells were allowed to adhere for 24 h before being re-fed every other day to allow for confluency. The cells were maintained in at 37 °C with an atmosphere of 5% CO_2_^52^. For treatment, after the seeded cells reached 70–80% confluency (about 12 h after seeding), exosomes were added. Each 30 mL whey pellet ultracentrifuged from 40–45 mL (27 ± 3 μg total RNA) of raw milk was mixed with 10 mL PBS and then filtered with 0.45 μm and 0.22 μm membranes for the treatment. Cells in 6-well plates were treated with 0.27 ± 0.03 μg total RNA/well, and 96-well plates were treated with 27 ± 3 ng and 55 ± 5 ng total RNA/well.

### MTT assay

The MTT assay has been confirmed to be feasible, rapid and reproducible. Moreover, its results have shown good correlation with those of other *in vitro* proliferation assays, such as the 3H-thymidine uptake assay^53^. For this study, the MTT kit was purchased from Beyotime Biotechnology (Shanghai, China) and used according to the manufacturer’s protocol. Briefly, IPEC-J2 cells were seeded in 96-well plates at the density of 5,000 cells per well with 200 μL of complete culture medium. After being allowed to adhere and spread for 12 h, the cells were treated with different concentrations of porcine milk exosomes for 24 h to 48 h. MTT assays were performed by incubating the exosome-treated IPEC-J2 cells with 20 μL (5 mg/mL) MTT labeling solution. After 4 h of incubation, IPEC-J2 cells were lyzed with 150 μL DMSO, and the purple formazan crystals were solubilized for detection at 570 nm^54^.

### CCK8 proliferation assay

The CCK8 kit was purchased from Beyotime Biotechnology and used according to the manufacturer’s protocol. IPEC-J2 cells were seeded and treated as described for the MTT method. After 24 h and 48 h, the supernatant was removed, and 100 μL of DMEM/F12 medium containing 10 μL of CCK8 was added to each well for incubation for another 3 h at 37 °C. The culture plates were then shaken for 10 min, and the OD values were read at 450 nm^55^.

### EdU assay

EdU (5-ethynyl-2′-deoxyuridine) is a labeled nucleoside analog of thymidine. In the S-phase of the cell cycle, its incorporation during DNA synthesis can reveal late replication regions^56^ and offers a wide range of opportunities to analyze cellular proliferation, population homeostasis and cell marking procedures^57^.

The following steps were carried out according to the manual of the Cell-Light™ EdU Apollo^®^488 *In Vitro* Imaging Kit (C10310-3) (Ribobio, Guangzhou, China). After treatment for 24 h or 48 h, 50 μM of the EdU labeling medium was added to the cell culture for incubation for 2 h at 37 °C with 5% CO_2_. Thereafter, cultured IPEC-J2 cells were fixed with 4% paraformaldehyde (pH 7.4, 50 μL/well of a 96-well plate) for 30 min and incubated with glycine (2 mg/mL) for 5 min. The cells were then washed with PBS (100 μL/well), and staining with anti-EdU working solution was performed at room temperature for 30 min. Following washing with 0.5% TritonX-100 (100 μL/well) in PBS for 5 min, the cells were incubated with 5 μg/mL Hoechst 33342 dye at room temperature for 30 min, followed by observation under a fluorescence microscope (OLYMPUS, Tokyo, Japan)^58^.

The percentage of EdU-positive cells was calculated from the Cell-Light™ EdU Apollo^®^488 *In Vitro* Flow Cytometry Kit (C10338-3) (Ribobio, Guangzhou, China) with slight modification of the EdU labeling procedure as follows. First, IPEC-J2 cells were seeded in a 6-well plate and collected after 24 h or 48 h of treatment by centrifugation at 1500 rpm/min for 5 min. After the supernatant was removed, the cells were resuspended with PBS and then centrifuged again at 1500 rpm/min for 5 min, followed by removal of the supernatant. Subsequently, the protocol of the Imaging Kit was followed, and at the last step, the percentage of EdU-positive cells was detected by flow cytometry with the Cytomics FC 500 MCL (Beckman Coulter, Brea, CA, USA) as previously reported^59^,^60^.

### Detection of expression of miRNAs and proliferation-related genes by qRT-PCR

IPEC-J2 cells were harvested after treatment for 48 h with porcine milk-derived exosomes and then used for RNA and protein extractions as follows. Total RNA was first digested with DNase I (Promega, Madison, WI, USA), and 2 μg of total RNA was reverse transcribed with oligo (dT). The cDNA was diluted 2-fold with ddH2O, and PCR was performed on a Bio-Rad system (Hercules, CA, USA) in a final 20 μL volume reaction, containing 2 μL PCR cDNA, 10 μL of 2× PCR Mix (Roche, Basel, Switzerland) and 1 mM of each primer. The real-time PCR thermal profile was as follows: 5 min at 95 °C, 40 cycles of 30 s at 94 °C, 30 s at the corresponding annealing temperature (Tm) and 72 °C for 30 s, followed by 72 °C at 10 min, and β-actin was used as an internal control for the PCR^19^,^61^. The milk-derived exosomes and IPEC-J2 miRNAs quantitative detected according to the protocol of Mir-X miRNA First Strand Synthesis Kit (Takara Bio Company, Dalian, China). The mRNA and miRNAs primers were designed with Primer 5.0 (Table 1).

### Western blotting

RIPA lysis buffer was used to extract IPEC-J2 cell proteins according to the assay kit protocol (Bioteke, Beijing, China). Briefly, 1 mM PMSF was added to the RIPA lysis buffer, and 100–200 μL was added to porcine milk exosomes. Following complete exosome lysis, the sample was centrifuged at 10,000–14,000 × *g* for 3–5 min, and the supernatant was subjected to further analysis. Proteins were stored at −80 °C until used.

Protein samples (20–30 μg) were measured by the BCA assay^62^ and separated using 10–15% SDS-PAGE, transferred to a 0.22 mm or 0.45 mm polyvinylidene difluoride membrane (Millipore, Bedford, MA, USA), incubated with specific and HRP-conjugated secondary antibodies, and detected with an enhanced chemiluminescence kit (Roche) using FluorChem M (ProteinSimple)^63^. Anti-p53 (D120082), anti-CDX2 (D162691) and anti-PCNA (D120014) antibodies were purchased from BBI Antibody (Sangon Biotech, Shanghai, China). IGF-1R and β-actin were purchased from Cell Signaling Technology (Danvers, MA, USA). Protein concentrations were determined using the Pierce BCA Protein Assay Kit (Thermo Fisher, Waltham, MA, USA) using a bovine serum albumin standard, and Image J software was used for gray scan analysis.

### Animal treatment

Male Kunming mice aged 18 days old were obtained from the Laboratory Animal Services Centre of Guangdong Province, China, and were kept in specific pathogen-free conditions. Sixty mice were evenly divided into five groups, including the control (supernatant), 0.125 μg, 0.25 μg, 0.5 μg and 1 μg total RNA dose groups (dissolved in 300 μL PBS). The time points for monitoring the mice were 2 and 3 weeks. Every day, each mouse was given the appropriate treatment by intragastric administration until to the end of experiment, at which time it was killed according to the guidelines of the Animal Experimentation Ethics Committee of South China of Agricultural University^64^. Three sections of duodenum and jejunum were collected. One section (3–5 cm) was fixed with 10% formalin for tissue sectioning, and the others were frozen in −80 °C until used for RNA and protein analysis.

### Intestinal histomorphology

Fixed intestinal tissues were taken for tissue sectioning and H&E staining. Sections were stained with H&E using standard pathologic procedures as previously reported^65^, including fixation in neutral buffered 10% formalin, embedding in paraffin and cutting into horizontal sections of 6 μm thickness. The villi height and crypt depth were determined under a light microscope (Nikon, Tokyo, Japan) at a magnification of 40× or 100×.

### qRT-PCR

Total RNA of intestinal tissue was extracted by TRIzol reagent (Invitrogen) as previously reported^66^, and the qRT-PCR analysis was carried out according to the above method for IPEC-J2 cells.

### Western blotting

Each tissue sample (0.5–1 μg) was ground in Fastprep-24 (MP Biomedicals, Santa Ana, CA, USA) with 300 μl RIPA lysis buffer according to the assay kit protocol (Bioteke) and then analyzed by Western blot according to the protocol used for IPEC-J2 cells.

### Statistical analysis

All data are expressed as the mean ± standard error of the mean (SEM). The significance of differences was determined using t-test for comparison of 2 groups, and one-way analysis of variance (ANOVA) with *post hoc* test of least significant difference (LSD) or Duncan test for multiple comparisons with SPSS 17.0. Differences were considered statistically significant at *P* < 0.05.

## Declarations

### Ethics Statement

The samples collected were according to the guidelines of Guangdong Province on the Review of Welfare and Ethics of Laboratory Animals approved by the Guangdong Province Administration Office of Laboratory Animals (GPAOLA). And the procedures were as the protocol of SCAU-AEC-2010-0416 approved by the Animal Ethics Committee of South China Agricultural University.

## Additional Information

**How to cite this article**: Chen, T. *et al.* Porcine milk-derived exosomes promote proliferation of intestinal epithelial cells. *Sci. Rep.* **6**, 33862; doi: 10.1038/srep33862 (2016).

## Supplementary Material

Supplementary Information

## Figures and Tables

**Table 1:** Primers for qRT-PCR.

Gene name	Primer	sequence (5′ to 3′)
*IGF-1R* (mouse)	IGF-1R -F	GGCAAGTATGCGTGAAAGAATC
	IGF-1R -R	CTAAAGGTCGGAGGAATGAGG
*PCNA* (mouse, pig)	PCNA -F	AGATGCCGTCGGGTGAAT
	PCNA -R	TCTCTATGGTTACCGCCTCCT
*p53* (mouse, pig)	P53-F	CATTGTCAGGCTTATGGAAACTAC
	P53-R	ACACTCGGAGGGCTTCACTT
*Cdx2* (mouse, pig)	Cdx2-F	ACCGCAGAGCCAAGGAGA
	Cdx2-R	AGGAGGTCACAGGAGTCAAGG
*β-actin* (mouse, pig)	Beta-actin -F	TGCTGTCCCTGTATGCCTCT
	Beta-actin	CTTTGATGTCACGCACGATTT
*IGF-1R* (pig)	IGF-1R -F	GAACCGCATCATCATCACC
	IGF-1R -R	CATCCTGCCCATCATACTCC
*FAS*(pig)	FAS-F	GATTTACCTGTATCGCTGGACC
	FAS-R	AGCAGAATGGACCCTCACG
*SERPINE*(pig)	SERPINE-F	CTACTTCTTCAGGCTGTTCCG
	SERPINE-R	AGGCAGTGGTGAGTGCTTTT
miR-7314		ATGCGGAACCTGCGGATAC
miR-1343		TATTATCTCCTGGGGCCCGC
miR-769-3P		CTGGGATCTCTGGGGTCTTGGTT
miR-181a		AACATTCAACGCTGTCGGTGAGTT
miR-128		TCACAGTGAACCGGTCTCTTT
miR-2320		TGGCACAGGGTCCAGCTGTCGG
