# Supplementary material for: Porcine milk-derived exosomes promote proliferation of intestinal epithelial cells
Source: Sci Rep. 2016 Sep 20;6:33862. doi: 10.1038/srep33862 (PMC5028765; doi:10.1038/srep33862)
Supplement: Supplementary Information [file srep33862-s1.pdf]

Porcine milk-derived exosomes promote proliferation of intestinal epithelial cells

Ting Chen §[1], Mei-Ying Xie §[1], Jia-Jie Sun[1], Rui-Song Ye[1], Xiao Cheng[1], Rui-Ping Sun[1], Li-Min Wei[1], Meng Li[1], De-Lin Lin[1], Qing-Yan Jiang[1], Qian-Yun Xi*[1], Yong-Liang Zhang*[1]

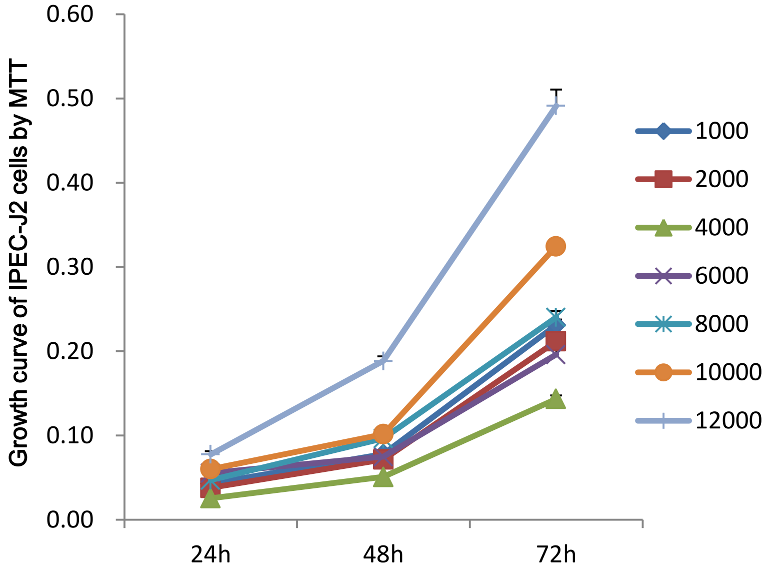

Supplementary Figure 1. Growth curve of IPEC-J2 cells by MTT assay. The OD value of 4,000–12,000 cells per/well displayed a good linear correlation at 48 h to 72 h after seeding (n=10).

100*(48h)

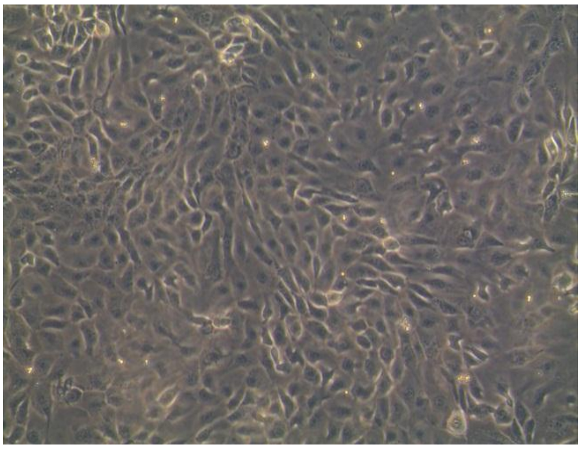

Exosomes (0.27 ±0.03 μg)

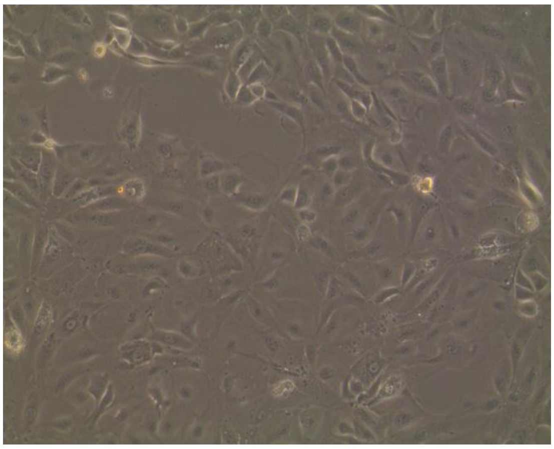

Control

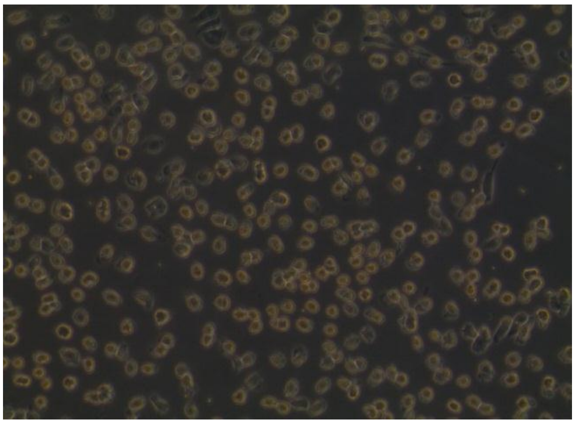

Exosomes (0.27 ±0.03 μg add digestive enzyme)

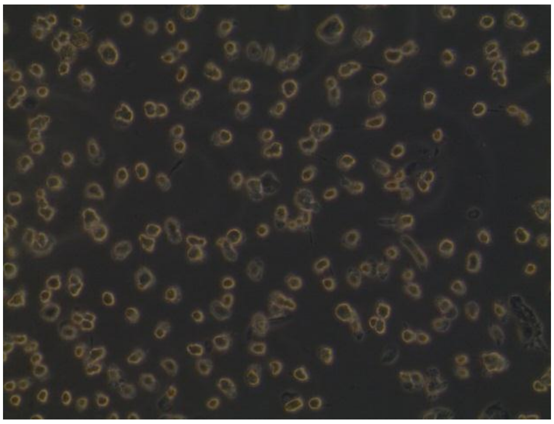

Control (add digestive enzyme)

Supplementary Figure 2. Microscopy examination of IPEC-J2 cell proliferation (27 ± 3 ng total RNA treatment group, 48 h). The number of IPEC-J2 cells was greater than the control group after treatment for 48 h with 55 ± 5 ng porcine milk exosome RNA.

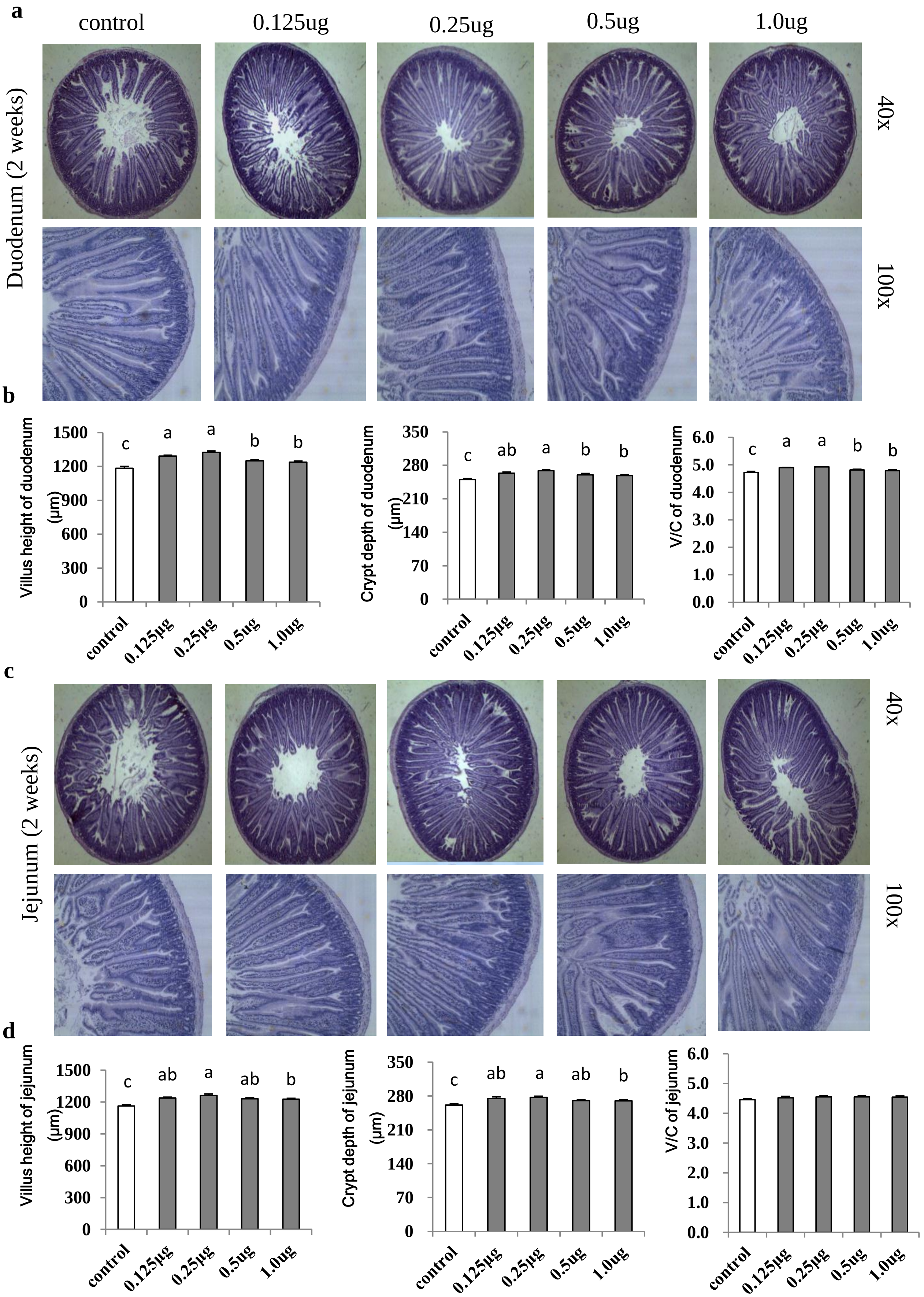
a
control
0.125ug
0.25ug
0.5ug
1.0ug
Duodenum (2 weeks)
40x
100x
b
Villus height of duodenum (μm)
1500
1200
900
600
300
0
c
a
a
b
b
control
0.125μg
0.25μg
0.5μg
1.0μg
Crypt depth of duodenum (μm)
350
280
210
140
70
0
c
ab
a
b
b
V/C of duodenum
6.0
5.0
4.0
3.0
2.0
1.0
0.0
c
a
a
b
b
c
Jejunum (2 weeks)
40x
100x
d
Villus height of jejunum (μm)
c
ab
a
ab
b
Crypt depth of jejunum (μm)
c
ab
a
ab
b
V/C of jejunum

Supplementary Figure 3. Intestinal histomorphology observation and analysis (2 weeks). (a) The 0.125 μg, 0.25 μg, 0.5 μg and 0.1 μg treatment groups showed improved villus height and crypt depth compared with the control group by microscopy observation of the duodenum morphology (n = 6). (b) Statistical analysis of the villus height, crypt depth and V/C ratio showed significant improvement in different treatment groups (duodenum, $P < 0.05$, n = 30). (c) The 0.125 μg, 0.25 μg, 0.5 μg and 0.1 μg treatment groups showed improved villus height and crypt depth compared with the control group by microscopy observation of the jejunum morphology (n = 6). (d) Statistical analysis of all treatment groups showed significantly increased villus height and crypt depth, but the V/C ratio was not significantly changed (jejunum, n = 30).
